# Characterization of Disease Patterns in Children with Intracranial Abscesses for Enhanced Clinical Decision-Making

**DOI:** 10.3390/pediatric16040085

**Published:** 2024-11-12

**Authors:** Maximilian Middelkamp, Marcus M. Kania, Friederike S. Groth, Franz L. Ricklefs, Lasse Dührsen

**Affiliations:** 1 Department of Neurosurgery, University Medical Center Hamburg-Eppendorf (UKE), 20246 Hamburg, Germany; F.Groth@uke.de (F.S.G.); f.ricklefs@uke.de (F.L.R.); L.Duehrsen@uke.de (L.D.); 2 Research Group Molecular Neurooncological Pathology, Center for Molecular Neurobiology Hamburg (ZMNH), University Medical Center Hamburg-Eppendorf, 20251 Hamburg, Germany; 3 Department of Pediatrics, University Medical Center Hamburg-Eppendorf, 20246 Hamburg, Germany; m.kania@uke.de

**Keywords:** brain abscess, central nervous system infections, intracranial infection, pediatric neurosurgery

## Abstract

Background: Intracranial suppurative infections in pediatric patients, while rare, pose a significant risk to patient mortality. Early recognition and fast initiation of diagnosis and treatment are crucial to prevent fatal outcomes. Between December 2022 and May 2023, a significant cluster of nine cases emerged, each necessitating neurosurgical intervention. This series highlights an important trend in clinical outcomes and raises questions about underlying factors contributing to this pattern. The need for surgical procedures in all instances suggests a commonality in severity, warranting further investigation into potential causes and preventative measures. This retrospective monocentric study aims to explore the clinical features associated with these cases to identify specific disease patterns that can expedite management in clinical practice. Methods: Cramer’s V effect size was employed to evaluate combinations of clinical features, followed by Fisher’s exact test applied to a constructed contingency table. A *p*-value was assessed for significance analysis, with combinations achieving a Cramer’s V value of 0.7 or higher being classified as exhibiting very strong correlations. Results: The analysis revealed distinct patterns of clinical features among children diagnosed with intracranial abscesses. Significant associations were identified, including correlations between sinusitis and Streptococcus pyogenes, and fever accompanied by affected temporal, frontal, and frontobasal lobe regions. Conclusions: Despite the generally limited statistical analysis of pediatric intracranial abscesses in the existing literature, this study provides meaningful significant associations between clinical features, delineating specific disease patterns for children with intracranial abscesses. By addressing this gap, the findings contribute valuable insights and offer a framework that could enhance clinical decision-making and support timely disease management in pediatric cases.

## 1. Introduction

Intracranial abscesses in children present significant clinical challenges due to their low incidence and nonspecific symptoms, with reported rates of approximately 0.3–1.8 per 100,000 inhabitants annually [1,2,3]. Recent changes in incidence have been noted, with a surge beginning in August 2021 and peaking during the winter of 2022–2023, following a period of reduced cases after COVID-19 lockdowns [4]. These life-threatening infections can arise from various risk factors, including specific infections, compromised immune systems, and head trauma [3,5]. Due to the diverse presentation of symptoms—ranging from neurological deficits and cephalalgia to altered mental status, seizures, and gastrointestinal disturbances such as nausea and vomiting—the timely diagnosis and management of intracranial abscesses in children remains difficult [1,2,6].

Diagnostic protocols typically involve a combination of laboratory investigations and neuroimaging techniques, particularly magnetic resonance imaging (MRI) or computed tomography (CT). The correct identification of the underlying pathogens—commonly streptococci, staphylococci, and anaerobic bacteria—can further complicate the diagnostic process [5,7]. Optimal management of these abscesses requires a comprehensive approach that includes anti-infective therapy, neurosurgical intervention, and supportive care. Conservative treatment options may be applicable for smaller or multiple abscesses, with a standard antibiotic regimen often including third-generation cephalosporins and metronidazole [6,8,9]. Furthermore, incorporating agents such as flucloxacillin or vancomycin may be essential for addressing potential methicillin-sensitive Staphylococcus aureus (MSSA) or methicillin-resistant Staphylococcus aureus (MRSA) [8]. The overall prognosis is contingent upon the severity of the infection, the presence of comorbid conditions, and the swiftness of treatment initiation [7].

Despite the existing literature, the current understanding of intracranial abscesses in pediatric populations remains inadequate, primarily due to the rarity of cases and the absence of robust clinical trials [10,11]. This study seeks to bridge this gap by conducting a retrospective analysis of nine pediatric cases of intracranial abscesses treated at a single center in Hamburg, Germany. The rationale was to derive statistic-based clinical characteristics from this specific patient cohort, thereby identifying specific disease patterns that may enhance clinical decision-making and facilitate expedited management strategies for this serious condition. This study provides meaningful significant associations between clinical features, delineating specific disease patterns for children with intracranial abscesses.

## 2. Materials and Methods

### 2.1. Patient Selection

**Figure 1:**
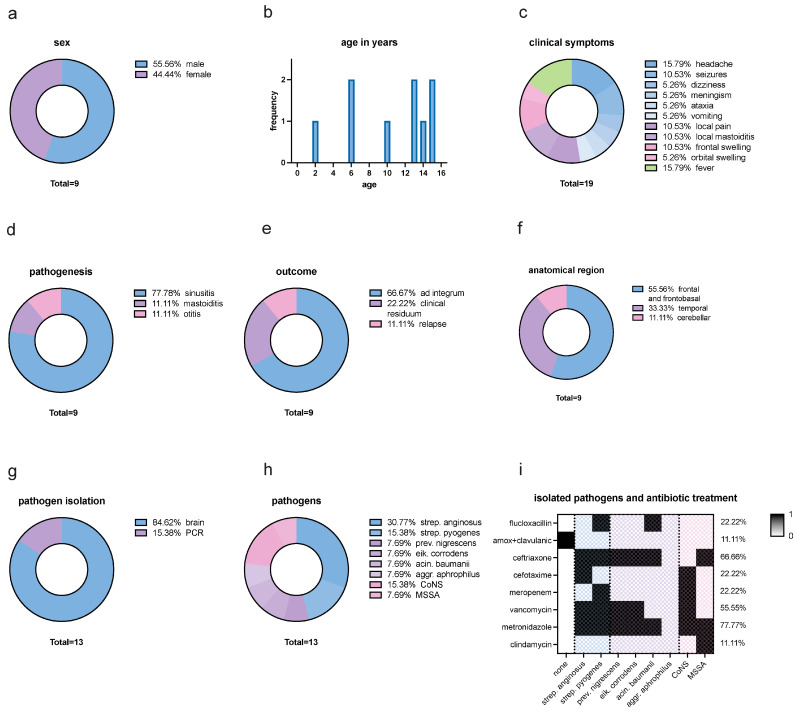
Descriptive Analysis. (**a**) Sex distribution: 55.56% were male and 44.44% female. (**b**) Age distribution in years: between the ages of 2 years to 15 years with a mean age of 10.22 years. (**c**) Clinical symptoms distribution: headache (15.79%), seizures (10.53%), dizziness (5.26%), meningism (5.26%), ataxia (5.26%), and vomiting (5.26%), which can be summarized as neurological symptoms. Further symptoms were local pain (10.53%) and local mastoiditis (10.53%), which can be summarized as local symptoms. Frontal swelling (10.53%), and orbital swelling (5.26%), which can be described as swelling symptoms, and fever (15.79%). Summarized, neurological symptoms make up 47.36% of all symptoms, while local symptoms contribute 21.06%. Swelling symptoms and fever both make up 15.79% of all symptoms. (**d**) Distribution of pathogenesis: 77.78% sinusitis, 11.11% mastoiditis, and 11.11% otitis. (**e**) Outcome: 66.67% of all cases showed an outcome ad integrum, 22.22% of cases had a clinical residuum such as mild flaccid paralysis of the left side and small scotoma superior et inferior on the right side, and 11.11% of all cases had a relapse. (**f**) Anatomical region: 55.56% were located frontal and frontobasal, 33.33% temporal, and 11.11% cerebellar (11.11%). (**g**) Isolation of pathogen material: 84.62% of the abscess material could be isolated from the brain and 15.38% by PCR. (**h**) Isolated pathogens: *Streptococcus anginosus* (30.77%), *Streptocuccus pyogenes* (15.39%), Coagulase-negative staphylococci (CoNS) (15.39%), Methicillin-sensitive *Staphylococcus aureus* (MSSA) (7.69%), *Prevotella nigrescens* (7.69%), *Eikenella corrodens* (7.69%), *Acinetobacter baumanii* (7.69%), and *Aggregatibacter aphrophilus* (7.69%). Hence, streptococci were the most common pathogens (46.15%), followed by anaerobic bacteria (30.76%) and staphylococci (23.07%). There was no case in which MRSA was isolated. (**i**) Isolated pathogens and antibiotic treatment: regarding all isolated pathogen results (none, mono- and polybacterial results), 88.88% of pathogens were treated with a third-generation cephalosporin, 77.77% with metronidazole, 55.55% with vancomycin, 22.22% with both meropenem and flucloxacillin, and 11.11% with both amoxicillin/clavulanic acid and clindamycin.

Through a digital data query from December 2022 to May 2023, all potential pediatric patients under the age of 18 years were identified using the complete set of codes from the International Classification of Diseases Version 10 (ICD-10) that pertain to intracranial infections and abscesses. To enhance reliability, a rigorous screening process was implemented, cross-verifying potential cases against clinical records. This involved manually reviewing diagnosis notes for accurate classification and identifying misclassifications. Any detected errors were reassessed using a standardized protocol, which included consultation with clinical experts and re-evaluation of diagnostic criteria. Cases involving meningitis and meningoencephalitis were excluded to focus specifically on neurosurgical patients where clear surgical interventions were indicated, producing a homogeneous study group. The relevant ICD-10 codes are presented in Table S1. Furthermore, to ensure the inclusion of any potentially misclassified patients, all individuals under the age of 18 who underwent neurosurgical procedures were also included in the study cohort. A total of nine cases of intracranial abscesses were analyzed, where one patient had two separate infections. Comprehensive epidemiological and clinical information was extracted from the digital patient’s chart, focusing on factors such as age, sex, clinical presentation, pathogenesis, pathogens, pathogen isolation, treatment regimen, and anatomical region for further investigation (Figure 1).

### 2.2. MRI

**Figure 2:**
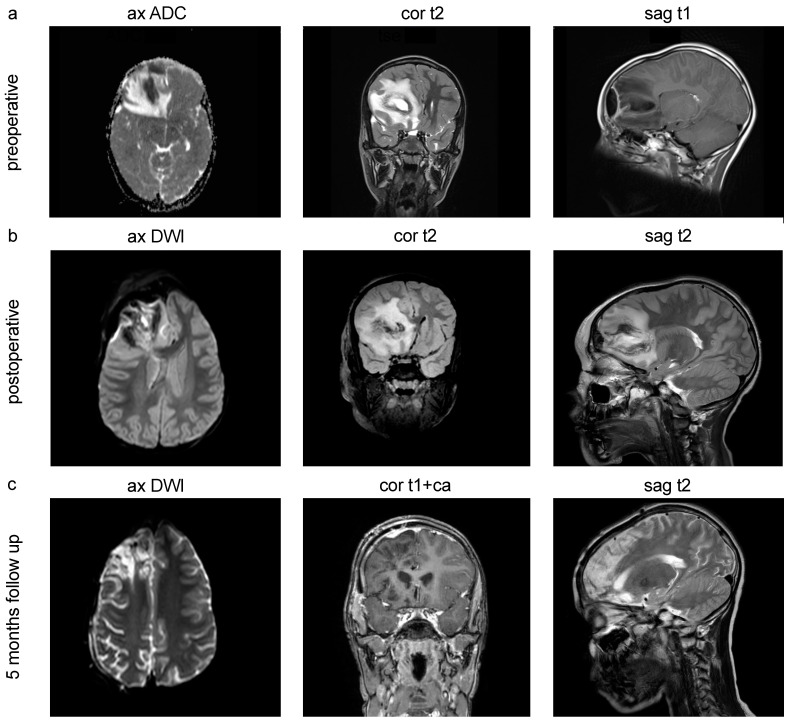
cMRI of pediatric intracranial abscesses. (**a**) Preoperative MRI scans of a 10-year-old male patient with pansinusitis and streptococcus anginosus infection, frontal and frontobasal intracranial brain abscess, midline shift subfalcine to the left, compressed right lateral ventricular anterior horn, and narrow sulcus drawing with stable spatial conditions. (**b**) Postoperative control MRI scans after abscess evacuation. (**c**) 5 months follow-up showing no delimitable hygromas or CSF leakage in the condition after neurosurgical hemicraniectomy and a known right hemispheric contrast enhancement. ax = axial, cor = coronal, sag = sagittal.

All cMRI scans were performed by a 3 Tesla magnetic resonance tomogram (Figure 2).

### 2.3. Statistics

**Figure 3:**
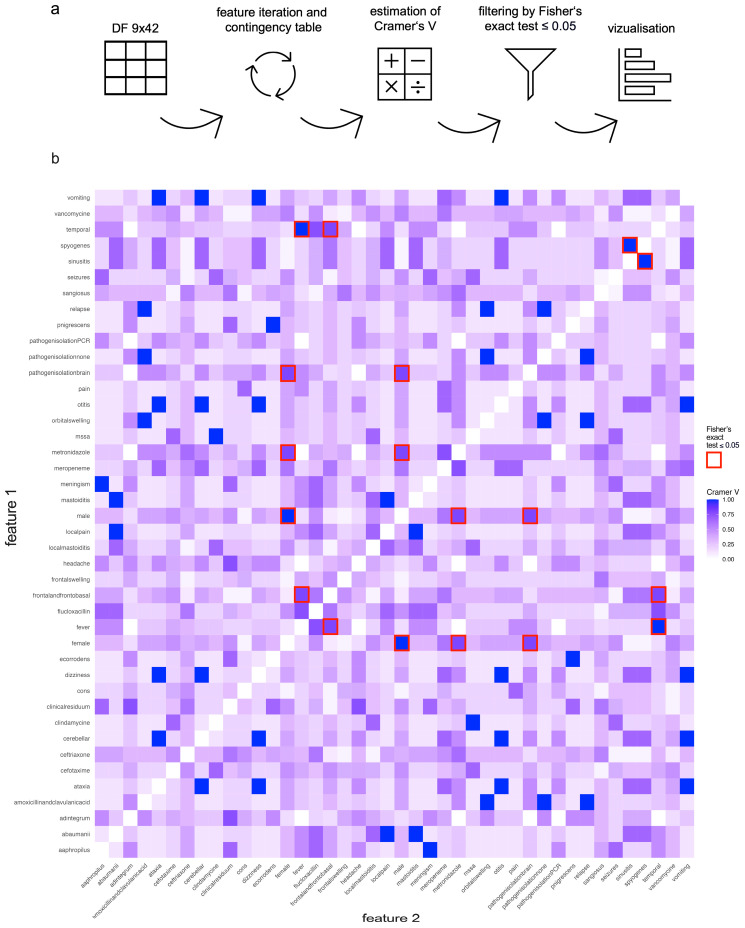
Statistical Feature Correlation Analysis. (**a**) Workflow of feature correlation analysis: The data were collected in a table and converted to a matrix nominal-binary coded as 0 (not present) and 1 (present). A code was created iterating over all combinations of two features. For each combination, a contingency table is created. Firstly, the Cramer’s V effect size was calculated for all combinations of two features. Secondly, Fisher’s exact test was performed on every contingency table. The *p*-value was checked for significance. After that, a heatmap correlation plot based on Cramer’s V value of every possible feature combination was created. (**b**) Statistical feature correlation analysis: 861 different contingency tables were created. A total of 36 feature combinations could be identified with a Cramer’s V value equal to or higher than 0.7, which indicates a very strong association. Testing on Fisher’s exact test afterward, all in all, nine feature combinations could be identified as significant with a very strong association: female vs. male (*p* = 0.007) sinusitis vs. streptococcus pyogenes (*p* = 0.028), fever vs. temporal (*p* = 0.012), frontal and frontobasal vs. temporal (*p* = 0.047), fever vs. frontal and frontobasal (*p* = 0.047), female vs. metronidazole (*p* = 0.047), female vs. pathogen isolation brain (*p* = 0.047), male vs. metronidazole (*p* = 0.047), and male vs. pathogen isolation brain (*p* = 0.047).

Descriptive statistics and data visualization were performed in GraphPad Prism 10. For statistical feature correlation analysis, RStudio 2022.07.2 + 576 was used and afterwards visualized by GraphPad Prism 10. The data were collected in a table and converted to a matrix nominal-binary coded as 0 (not present) and 1 (present) and all combinations of the two features were iterated. For each combination, a contingency table was created. First, the Cramer’s V effect size was calculated for all combinations of two features (Table S2). Then, Fisher’s exact test was performed on the contingency table (Table S3). The *p*-values ≤ 0.05 were considered statistically significant (Figure 3a). Fisher’s exact test was chosen for its suitability for small sample sizes. Unlike the Chi-Square Test, which requires a minimum expected frequency that is often difficult to meet in smaller datasets, Fisher’s exact test provides precise probability calculations without these limitations. This ensures the validity of our results, particularly when dealing with rare events. Feature combinations with a Cramer’s V value equal to or higher than 0.7 are considered a very strong correlation. Cramer’s V is a measure of association between two categorical variables, ranging from 0 to 1. A value of 0 indicates no association, while a value closer to 1 suggests a stronger association. Specifically, a Cramer’s V value of 0.7 indicates a strong link between the variables, implying that changes in one variable are closely related to changes in another [12,13]. After that, a correlation plot based on Cramer’s V value of every possible feature combination was created (white = 0, blue = 1). Significant feature combinations of Fisher’s exact test were highlighted with a red rectangle (Figure 3b).

## 3. Results

### 3.1. Epidemiology

In this monocentric, retrospective study from December 2022 to May 2023, nine cases of intracranial infections were recorded in eight pediatric patients, where one patient had two separate infections, at the University Medical Centre Hamburg-Eppendorf (UKE) in Hamburg, Germany. All patients were treated at the Department of Pediatrics and underwent neurosurgical treatment for abscess evacuation and sample collection. Investigating all cases, 55.56% were male and 44.44% female (Figure 1a), and patients were between the ages of 2 to 15 years, with a mean age of 10.22 years (Figure 1b).

### 3.2. Clinical Symptoms

At the initial clinical presentation, a range of diverse symptoms was observed, including headache (15.79%), seizures (10.53%), dizziness (5.26%), meningism (5.26%), ataxia (5.26%), and vomiting (5.26%), which can be summarized as neurological symptoms. Further symptoms were local pain (10.53%) and local mastoiditis (10.53%), which can be summarized as local symptoms, and frontal swelling (10.53%), orbital swelling (5.26%), and fever (15.79%). Neurological symptoms make up 47.36% of all symptoms, while local symptoms contribute 21.06%. Swelling symptoms and fever both make up 15.79% of all symptoms (Figure 1c). In terms of pathogenesis, the continuous spread from a preceding infection occurred in 77.78% of cases of sinusitis, 11.11% of cases of mastoiditis, and 11.11% of cases of otitis (Figure 1d). Regarding the outcome, 66.67% of all cases showed an outcome ad integrum, 22.22% had a clinical residuum such as mild flaccid paralysis of the left side and small scotoma superior et inferior on the right side, and 11.11% of all cases had a relapse (Figure 1e).

### 3.3. Treatment

As soon as an intracranial abscess was suspected, a cMRI imaging was performed. After an interdisciplinary case discussion between pediatricians and neurosurgeons, a prompt neurosurgical treatment was performed within 24 h. The abscess formation was microsurgically evacuated and sent for microbiological and histopathological diagnosis. Abscess drainage was not used in any case. Extubation was performed promptly in the pediatric intensive care unit, followed by starting the empirical antibiotic treatment until the pathogen-specific antibiogram was available.

In nine cases, a total of 13 different pathogens could be isolated. Regarding the intracranial anatomical abscess region, 55.56% were located in the frontal and frontobasal regions (Figure 2), 33.33% in the temporal region, and 11.11% in the cerebellar region (11.11%) (Figure 1f). Pathogen material was isolated in 84.62% of all cases using abscess material from the brain and in 15.38% was isolated by PCR (Figure 1g). In one case, no pathogens could be isolated. Blood cultures were sterile in all cases. The isolated pathogens are *Streptococcus anginosus* (30.77%), *Streptocuccus pyogenes* (15.38%), Coagulase-negative staphylococci (CoNS) (15.38%), Methicillin-sensitive *Staphylococcus aureus* (MSSA) (7.69%), *Prevotella nigrescens* (7.69%), *Eikenella corrodens* (7.69%), *Acinetobacter baumanii* (7.69%), and *Aggregatibacter aphrophilus* (7.69%) (Figure 1h). Hence, streptococci were the most common pathogens (46.15%), followed by anaerobic bacteria (30.76%) and staphylococci (23.07%). There was no case in which MRSA was isolated.

### 3.4. Etiology

In 66.66% of all cases, a third-generation cephalosporin was administered along with metronidazole. In 44.44% of all cases, flucloxacillin or vancomycin was added to the regimen. One patient received clindamycin additionally. The patient who was readmitted with a relapsed infection was treated with amoxicillin/clavulanic acid in the first episode. Meropenem in combination with vancomycin was initiated in 22.22% of all cases. The empirical anti-infective therapy demonstrated an efficacy of 77.77% against the identified pathogens. In the initial episode of the patient who had a reinfection, the detection of a causative pathogen was not possible. Retrospectively, with the isolation of Strep. anginosus, the insufficient antibacterial activity of amoxicillin/clavulanic acid may be considered. Looking at all isolated pathogen results (none, mono- and polybacterial results), 88.88% of pathogens were treated with a third-generation cephalosporin, 77.77% with metronidazole, 55.55% with vancomycin, 22.22% with both meropenem and flucloxacillin, and 11.11% with both amoxicillin/clavulanic acid and clindamycin (Figure 1i).

### 3.5. Statistical Feature Correlation Analysis

To analyze the various features of all cases and identify them, if there is a significant association between two features, Cramer’s V test and Fisher’s exact test were performed. With Cramer’s V test, one can measure the strength of the relationship between two categorical variables. While V = 0 displays a weak association, V = 1 indicates a strong association without giving a statement on the direction. Fisher’s exact test is then used to examine the significancy of measured associations between two categorical variables, especially with a small number of cases. Figure 3a shows the workflow. With 42 different features, the number of feature combinations excluding same feature comparison is 861, leading to an individual contingency table for each feature combination (Table S2). A total of 36 feature combinations could be identified with a V value equal to or higher than 0.7, which indicates a very strong association [12,13]. Using Fisher’s exact test, all in all, nine feature combinations could be identified as significant. All of them display a Cramer’s V value higher than 0.7, indicating a very strong association: female vs. male (*p* = 0.007), sinusitis vs. streptococcus pyogenes (*p* = 0.028), fever vs. temporal (*p* = 0.012), frontal and frontobasal vs. temporal (*p* = 0.047), fever vs. frontal and frontobasal (*p* = 0.047), female vs. metronidazole (*p* = 0.047), female vs. pathogen isolation brain (*p* = 0.047), male vs. metronidazole (*p* = 0.047), and male vs. pathogen isolation brain (*p* = 0.047) (Figure 3b, Table S3).

### 3.6. Recommendations for Clinical Decision-Making

For day-to-day clinical decision-making, the correlation between sinusitis and Streptococcus pyogenes highlights the necessity for increased clinical awareness. Recognizing sinusitis as a risk factor for these serious intracerebral infections may encourage clinicians to adopt more proactive diagnostic strategies, such as lowering the threshold for MRI in children with sinusitis and concerning neurological symptoms. This awareness could also lead to earlier consideration of targeted antibiotic therapy against Streptococcus pyogenes. Implementing these changes in diagnostic and treatment protocols could facilitate earlier intervention, potentially preventing progression to severe complications like brain abscesses that may require emergency surgery.

## 4. Discussion

### 4.1. Coherent Clinical Presentation of Children with Intracranial Abscesses with a Streptococcal and Anaerobic Pathogen Spectrum

In this section, the treatment regime used in this study will be compared with the current literature. Intracranial abscesses in children are a rare and potentially fatal disease. Knowledge about clinical, diagnostic, and therapeutic parameters is important for optimal treatment. In our study, we could not find a difference in sex. The mean age of 10.2 years aligns with previous research [14]. Regarding the distribution of clinical symptoms, we found that half of the clinical symptoms were neurological, while the remaining half were split between swelling, fever, and local symptoms, suggesting that the occurrence of these symptom complexes should be present in the differential diagnosis of intracranial abscess [15,16]. The prevalence of streptococci and staphylococci mirrors the distribution observed in previous studies. Moreover, this study also found a higher prevalence of anaerobes in pediatric intracranial abscesses, which is consistent with new findings from the existing literature [17,18]. It can be strongly suspected that pathogen patterns in brain abscesses can exhibit regional variations, influenced by factors such as healthcare practices, demographics, and environmental conditions. For instance, Bodilsen et al. (2024) found that oral cavity bacteria, particularly the *Streptococcus anginosus* group, *Fusobacterium* spp., and *Aggregatibacter* spp., are the most common pathogens in community-acquired brain abscesses, often linked to dental and ear infections. While less common pathogens include *Staphylococcus aureus*, Gram-negative bacilli, and *Mycobacterium tuberculosis*, many contributing factors, such as dental infections and immunodeficiency, have played significant roles in recent decades. Emerging molecular diagnostics have improved pathogen identification, revealing a high prevalence of anaerobic bacteria consistent with our findings [17,19]. Moreover, while specific literature on pediatric brain abscesses and climate change is limited, there is broader recognition of climate change’s impact on infectious diseases. Social, demographic, and financial factors also significantly influence health outcomes and can lead to disparities in access to diagnostics and treatment. The COVID-19 pandemic has further exacerbated these issues, affecting healthcare availability for children and highlighting the importance of understanding these dynamics to ensure equitable healthcare access for all populations [19,20].

### 4.2. Sinusitis as the Primary Cause of Intracranial Abscesses in Children: Successful Outcomes with Neurosurgical Craniotomy and Antibiotic Treatment Resulting in No Fatalities

Based on our study, the high prevalence of sinusitis with the above-mentioned pathogens as the underlying cause of the development of intracranial infections goes in line with the current literature [14,21]. Moreover, acute mastoiditis can also cause serious intracranial complications, with 10% of brain abscess cases being linked to it in our study. In these cases, the literature recommends a timely intervention by mastoidectomy, with higher success rates compared to an antibiotic treatment [22]. When examining the timing of these intracranial abscess cases, it is noteworthy that they increased following the relaxation of COVID-19 pandemic restrictions. This may suggest that the reduced transmission of respiratory pathogens during the pandemic, including bacteria and viruses causing sinusitis, may have influenced the rise in intracranial infections observed in our study period. This could represent a rebound effect, which might have implications for the targeted treatment and prevention of sinusitis and its complications. In terms of the affected anatomical areas, our findings show that half of the infections occurred in the frontal region and a third occurred in the temporal region. This distribution likely reflects sinusitis as a predominant pathway for infection. [14,21]. Thus, the ability to isolate pathogens in a highly significant proportion of cases using material from surgery suggests that access to proper diagnostic procedures appears to be a reliable method to compare our studies (84%) to the known literature (60–89%) [23,24]. Moreover, the use of third-generation cephalosporin and metronidazole as empiric antibiotic treatments in most cases appears to be effective [5,25]. The continuation of antibiotic treatment after surgery was tailored based on the specific recommendations of the in-house infectious disease guidelines and antibiotic stewardships and varied accordingly. The guidelines emphasize the importance of antibiotic stewardship in brain abscess treatment, focusing on the appropriate duration of therapy and judicious use of oral antibiotics. The optimal duration balances relapse risk with toxicity concerns and stewardship principles, with the guidelines recommending 6–8 weeks of intravenous antimicrobials for most cases but acknowledging the potential for shorter durations in specific circumstances. The lack of definitive evidence supporting early oral transition highlights the need for cautious antibiotic use and further research in this area [17].

Furthermore, in the cases presented, abscess drainage was not employed, despite being a surgical treatment option used in other neurosurgical centers. The decision to forego abscess drainage was based on several considerations. Firstly, the available literature does not conclusively demonstrate that abscess drainage is superior to radical evacuation in pediatric patients. On the one hand, craniotomy and evacuation may reduce the need for additional imaging, surgeries, and antibiotic therapy, leading to more streamlined patient management and fewer complications [26]. On the other hand, the Double-Cavity Sleeve Tube drainage approach combines the advantages of smaller burr hole techniques, providing a less invasive treatment option that not only simplifies the procedure but also enhances patient safety [27]. Our cases involved acute, large, space-occupying abscesses that presented a potentially life-threatening scenario. In such circumstances, radical intervention through craniotomy and evacuation was deemed necessary to address the urgency and severity of the clinical presentation. Nevertheless, there is a lack of follow-up studies specifically addressing this issue in pediatric patients. Following the mentioned treatment regime, most cases in our analysis showed a favorable outcome (resitutio ad integrum) without a death event and a significant reduction in the follow-up cMRI (Figure 2c).

### 4.3. Feature Analysis Reveals Clinical Feature Combinations to Be Considered for Early Diagnostics and Treatment

Our study showed a statistically significant strong association between both male and female sexes, suggesting that the occurrence of intracranial abscesses in children is independent of gender. Additionally, a strong association was observed between the clinical symptoms of sinusitis and the pathogen Streptococcus pyogenes, indicating that in such cases, the early consideration of an intracranial abscess is crucial and may be diagnosed by early imaging, preferably with cMRI [28]. Additionally, a statistically significant strong correlation was found between the clinical symptoms of fever and the anatomical presence of the abscess in the temporal and frontal/frontobasal regions. Consequently, fever serves as a crucial clinical marker for abscess formation in the frontal and temporal areas [29]. Successful pathogen isolation from surgery was also found to be statistically significantly associated with both male and female pediatric patients, implying that this is independent of gender. Moreover, the use of metronidazole was identified as an important component of the antibiotic therapy, showing a statistically significant strong association in both male and female patients.

### 4.4. Decreased Overall Transmission of Respiratory Pathogens During COVID-19 Pandemic Maybe Led to a Shift in Incidence and a Seasonal Increase in Intracerebral Abscesses in Children Due to a Catch-Up Effect

During the pandemic, the implementation of strict rules and guidelines, such as social distancing, wearing masks, and increased emphasis on hand hygiene, aimed to reduce the transmission of not only the COVID-19 virus but also other respiratory pathogens [30]. Most of the COVID-19 measures in Hamburg and Schleswig-Holstein ended with the expiry of the hotspot regulation in April 2022. These preventive measures could have resulted in a decreased overall transmission of respiratory pathogens, including those commonly associated with sinusitis [31]. Nevertheless, physiologically, infections build up immunity, which protects against re-infection by the same pathogen, at least for a certain period, which is called adaptive immune response. This protection wears off again after a few years [32]. The easing of hygiene measures may result in a resurgence of infections, as many children have grown more vulnerable to respiratory pathogens after a prolonged period without exposure. Cohen et al. published a highly discussed hypothesis in 2021 that the lack of immune stimulation due to the reduced circulation of microbial agents may induce an “immunity debt”, which could have negative consequences when the pandemic was under control and non-pharmaceutical interventions (NPIs) are lifted [33,34]. Nevertheless, the occurrence of intracranial infections in children necessitates additional research and analysis. Factors such as changes in healthcare-seeking behavior, delayed diagnoses, or differences in the presentation of symptoms during the pandemic may also contribute to the observed patterns. Therefore, additional research and data collection are needed to fully understand whether reduced exposure to pathogens has an impact on the increased development of intracranial abscesses in children.

### 4.5. Close Follow-Up Examinations for Children with Neurological Deficits After Early Intervention of Intracranial Abscesses May Require an Individualized Rehabilitation Plan to Improve Outcomes

In this study, the association between the presence and severity of residual symptoms, such as left-sided paralysis, and the location of the abscess along with affected cerebral compartments was acknowledged. The criticality of timely intervention (“time is brain”) emphasizes that treatment delays can exacerbate neurological deficits. Rapidly occurring complications, including space-occupying lesions, midline shift, elevated intracranial pressure, and herniation, may result in adverse outcomes, potentially leading to death. Current studies indicate that approximately up to 70% of adult survivors of intracranial abscesses demonstrate neurological deficits, with significant long-term effects on employment and disability pensions. In children, sequelae can severely impact daily life, education, and social interactions, leading to considerable long-term quality-of-life repercussions. Therefore, the crucial role of specialized neurorehabilitation in regaining functional capacity is emphasized, supporting the need to further analyze the long-term consequences of residual symptoms. Understanding these potential sequelae is essential for informing prognosis and tailoring rehabilitation efforts. Early intervention strategies, focusing on physical and occupational therapy, may enhance recovery of motor function and overall quality of life. Ongoing neuropsychological assessment remains important for addressing cognitive and emotional difficulties that may arise post-treatment [17].

### 4.6. Strengths and Limitations

Several notable strengths are present in this study, which enhance the reliability and validity of the findings. Firstly, a meticulous collection of clinical data was conducted, ensuring that the information gathered was comprehensive and accurate. Additionally, advanced statistical methods, including Cramer’s V and Fisher’s exact test, were employed to explore the correlations within the dataset. This approach not only allows for the quantification of the strength of associations between variables but also adds depth and nuance to the analysis, providing a greater understanding of the relationships examined. Nevertheless, several limitations of the study must be acknowledged, which impact the interpretation of the findings. Firstly, a retrospective design was employed, which introduces potential biases related to data collection and interpretation, thereby limiting the ability to establish causal relationships. Additionally, the reliance on medical records for data extraction may lead to data collection bias, as the accuracy and completeness of these records can vary. Consequently, any instances of missing or incomplete data may influence the results. Moreover, the study population consisted of patients drawn from a specific clinical setting, raising concerns about selection bias and limiting the generalizability of the findings to broader populations or different healthcare environments. Finally, the statistical analyses were constrained by a small sample size, which may affect the power to detect significant differences or associations and raise the possibility of important findings being overlooked. This study provides valuable insights into the associations and clinical characteristics of pediatric patients with intracranial abscesses. To overcome the mentioned limitations, further research is needed, particularly in larger, multicenter studies. Additionally, investigating potential confounding factors and incorporating longitudinal follow-up would strengthen the validity of these findings. However, with this study, we have made a statistical contribution to the treatment regimen for intracranial abscesses in children, an area where there is only limited data available.

## 5. Conclusions

Taken together, we had no death events and approximately 22% of patients had clinical residuals in our study. Given the severity of this condition, immediate imaging, ideally with cMRI, is essential in cases where there is clinical suspicion based on symptoms like sinusitis, fever, and neurological issues. Prompt surgical abscess evacuation should be aimed for, and postoperative calculated antibiotic therapy should be initiated immediately following the antibiogram. Here, we show significant associations such as sinusitis and Streptococcus pyogenes of children with intracranial abscesses, revealing a specific disease pattern that could be useful in clinical practice for decision-making and fast disease management. In the future, utilizing rapid PCR for pathogen identification may be promising, as it could enhance accuracy and speed in diagnostics, leading to more targeted treatment and timely therapy. Also, follow-up assessments and long-term outcome evaluations for children with residual neurological effects should be performed to gather valuable data on neurological outcomes. Further research is needed to expand upon these findings.

## Data Availability

The original contributions presented in the study are included in the article/Supplementary Materials; further inquiries can be directed to the corresponding author.

## Supplementary Materials

The following supporting information can be downloaded at https://www.mdpi.com/article/10.3390/pediatric16040085/s1, Table S1: ICD10 Codes; Table S2: Cramer’s V test, Table S3: Fisher’s *t*-test.
